# A Brief Review on Additive Manufacturing of Polymeric Composites and Nanocomposites

**DOI:** 10.3390/mi12060704

**Published:** 2021-06-16

**Authors:** Vahid Monfared, Hamid Reza Bakhsheshi-Rad, Seeram Ramakrishna, Mahmood Razzaghi, Filippo Berto

**Affiliations:** 1 Department of Mechanical Engineering, Zanjan Branch, Islamic Azad University, Zanjan, Iran; 2 Advanced Materials Research Center, Department of Materials Engineering, Najafabad Branch, Islamic Azad University, Najafabad, Iran; mahmood.razzaghi@gmail.com; 3 Department of Mechanical Engineering, National University of Singapore, 9 Engineering Drive 1, Singapore 117576, Singapore; seeram@nus.edu.sg; 4 Department of Mechanical and Industrial Engineering, Norwegian University of Science and Technology, 7491 Trondheim, Norway

**Keywords:** additive manufacturing, polymeric matrix composite, mechanical properties

## Abstract

In this research article, a mini-review study is performed on the additive manufacturing (AM) of the polymeric matrix composites (PMCs) and nanocomposites. In this regard, some methods for manufacturing and important and applied results are briefly introduced and presented. AM of polymeric matrix composites and nanocomposites has attracted great attention and is emerging as it can make extensively customized parts with appreciably modified and improved mechanical properties compared to the unreinforced polymer materials. However, some matters must be addressed containing reduced bonding of reinforcement and matrix, the slip between reinforcement and matrix, lower creep strength, void configurations, high-speed crack propagation, obstruction because of filler inclusion, enhanced curing time, simulation and modeling, and the cost of manufacturing. In this review, some selected and significant results regarding AM or three-dimensional (3D) printing of polymeric matrix composites and nanocomposites are summarized and discuss. In addition, this article discusses the difficulties in preparing composite feedstock filaments and printing issues with nanocomposites and short and continuous fiber composites. It is discussed how to print various thermoplastic composites ranging from amorphous to crystalline polymers. In addition, the analytical and numerical models used for simulating AM, including the Fused deposition modeling (FDM) printing process and estimating the mechanical properties of printed parts, are explained in detail. Particle, fiber, and nanomaterial-reinforced polymer composites are highlighted for their performance. Finally, key limitations are identified in order to stimulate further 3D printing research in the future.

## 1. Introduction

Nowadays, AM is a highly discussed topic and subject in scientific and industrial societies and is a new vision to the unknown modern world, as it is considered the fifth industrial revolution. Updated special academic investigations in AM and 3D printing fields are very limited particularly a small encyclopedia of AM and obtained results. AM materials’ future involves essential changes such as employing parallel methods and technologies. Considerable and significant developments have been obtained in the fields of four-dimensional (4D) and five-dimensional (5D) printing based on AM or 3D printing techniques. Some possibilities for the future of AM technologies may lie in the medical sciences and tissue engineering [1,2]. Additionally, AM enables the development of drug delivery systems with tailored forms, sizes, colors, and tastes for patient choice, thereby increasing patient compliance [3,4,5]. Recently, researchers have investigated AM for the release of multiple medicines from a single tablet (multilets) and to attain a programmed drug release profile using a combination of Fused deposition modeling (FDM) and injection molding [6], as well as for directly printing certain structures [7,8]. These structures may be easily adjusted to obtain the desired release profile based on the drug’s characteristics and physiological and environmental conditions. Pharmaceutical substances must be released based on knowledge of the underlying release processes and their specific properties [9]. For example, AM may be beneficial in challenges posed by COVID-19. Besides, it is predictable that AM will create innovative materials with new technologies in the near future. The study on AM is vital and necessary for generating the subsequent developments that are beneficial and vital for human beings and life [1,2]. This research article presents a concise review of AM, polymeric composites, and applied results published in recent years. Table 1 and Table 2 present the nomenclature and abbreviation used in this research article.

Compared to conventional subtractive manufacturing methods, AM, or 3D printing, it is characterized as adding materials layer by layer to manufacture constructs based on the computer-aided designed (CAD) 3D models. In over more than three decades of AM development, many novel AM methods have been introduced in different applications, including medicine, aerospace, tissue engineering, automotive, biomedical, and architectural design [1,2]. Recently, significant advances have been made in the 3D printing of polymer matrix fiber-reinforced composites (FRCs) and possible design and analysis methods for these 3D printed structures. To consider the challenges in AM of polymer matrix composites (PMCs) reinforced with fibers, the most recent developments and improvements to existing methods have been analyzed thoroughly [2].

**Figure 1:**
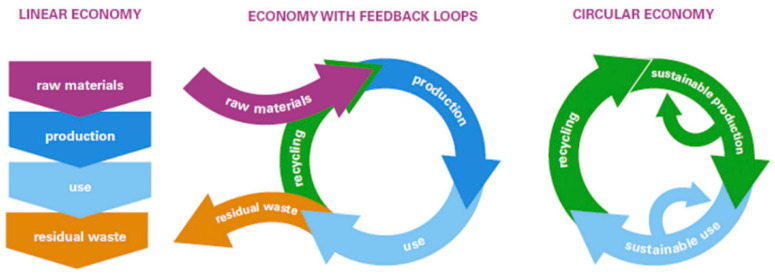
Life cycle stages in the linear, recycling and circular economy models [4,10].

**Figure 2:**
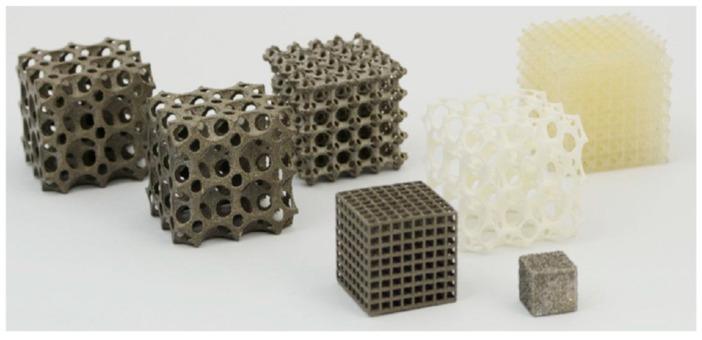
Examples of 3D scaffold structures manufactured using AM technology [11].

The rapid progress in AM of polymeric composites has paved the way for a modern circular economy focused on mass production and distributed recycling. Circular economy “circularity” is an economic process and structure aimed at eliminating waste and ensuring the continued use of resources, as illustrated in Figure 1 [4,10]. The concept of distributed recycling for additive manufacturing (DRAM) refers to using recycled materials in the process chain of 3D printing through a mechanical recycling process. The circular economy has the potential to reduce material, waste, and manufacturing costs significantly [10]. Many advantages of AM process make it one of the most suitable routes for fabricating complicated scaffold structures. Figure 2 illustrates examples of AM processes 3D constructs in the form of scaffolds [11].

**Figure 3:**
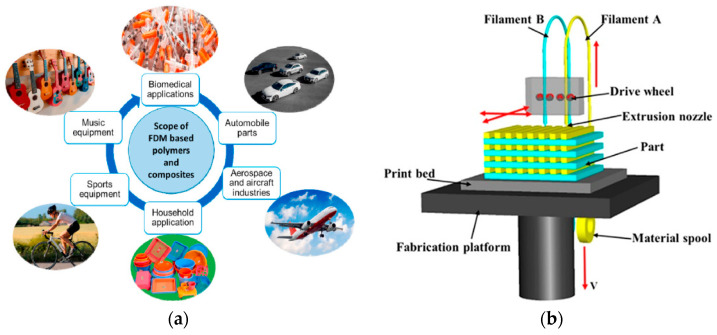
(**a**) Applications of FDM-based polymers and PMCs [12], and (**b**) schematic representation of a typical FDM setup [14].

FDM is a flexible AM technique used for 3D printing of polymers and PMCs. To build a 3D object, material is deposited using a computer-controlled 3D printer in successive layers. FDM-based polymer research has been increased in recent years because of its versatility in developing polymers and PMCs [12]. FDM-based polymers have the high potential to be utilized in the diverse applications; Figure 3a depicts the numerous ranges of FDM-based polymers in several applications [12]. FDM produces low-cost components with an acceptable surface finish and high durability. The raw material for FDM is often wire or filament with a diameter of 2.85 mm or 1.75 mm, depending on the 3D printer utilized [13]. As can be seen in Figure 3b [14], FDM printers operate by the controlled extrusion of thermoplastic filaments. In an FDM process, filaments melt into a semi-liquid state at the nozzle and are extruded layer by layer onto the build platform, where they fuse and solidify into final specimens [14].

**Figure 4:**
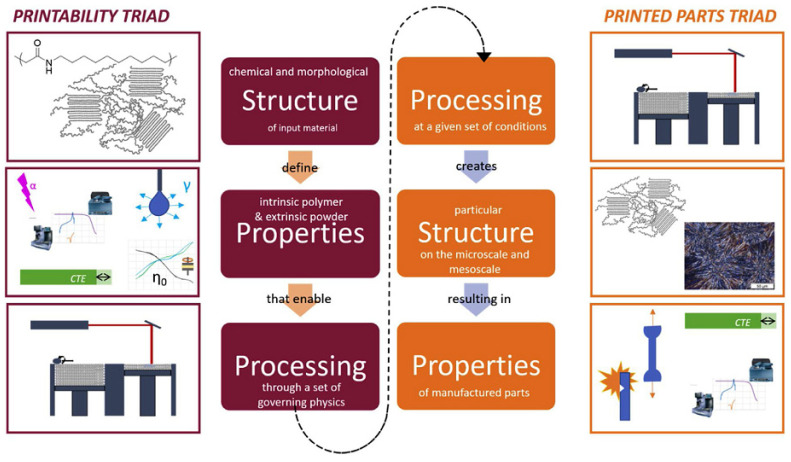
The two, key relationship triads of polymer PBF manufacturing [22].

FDM has been used to manufacture polymer-based fiber composites in recent years [15]. Incorporating fiber into the thermoplastic matrix resulted in an increase in modulus, tensile strength, and bending strength compared to the pristine thermoplastic material [2,16]. This enhances the likelihood of FDM-printed materials being used in load-bearing applications. However, uncertainties associated with the FDM manufacturing process, such as the formation of voids, defects, and inefficient layer bonding, increase the likelihood of polymer and composite failure [17]. Regardless of the benefits of FDM, the material performance is critical in determining the materials’ durability and reliability. The performance of FDM components is affected by a variety of parameters, including the following: (i) the effect of printing parameters; (ii) the effect of bonding characteristics; (iii) the effect of material and reinforcement; and (iv) the effect of the FDM process faults. All of these aspects contribute significantly to the mechanical strength of 3D printed fiber composites [18]. Powder bed fusion (PBF) is the most often used AM method for the fabrication of load-bearing biomaterials [19]. The layer thickness on the platform is generally 100 µm for polymer powders and 20 to 100 µm for metal and ceramic powders in laser-based processes such as selective laser sintering (SLS), selective laser melting (SLM), and direct metal laser sintering (DMLS). However, electron beam-based PBF (i.e., electron beam melting, EBM) commonly uses a layer thickness of 50 to 200 µm [20,21]. There are two sets of relationships to be considered for any fabricating process downstream of polymerization, such as PBF: (1) the interactions between the input polymer structure and properties and the manufacturing technology that enables successful production (“printability triad”), and (2) the effect of processing conditions on the obtained microstructure and properties of manufactured parts. Figure 4 illustrates these two triads graphically for polymer PBF manufacturing [22]. The triads describe two unique aims of fundamental polymer manufacturing science: (1) fabricating a component with PBF and (2) fabricating a component with specific attributes using PBF. Transitioning between the triads necessitates a paradigm change for specific polymer characteristics [22].

**Figure 5:**
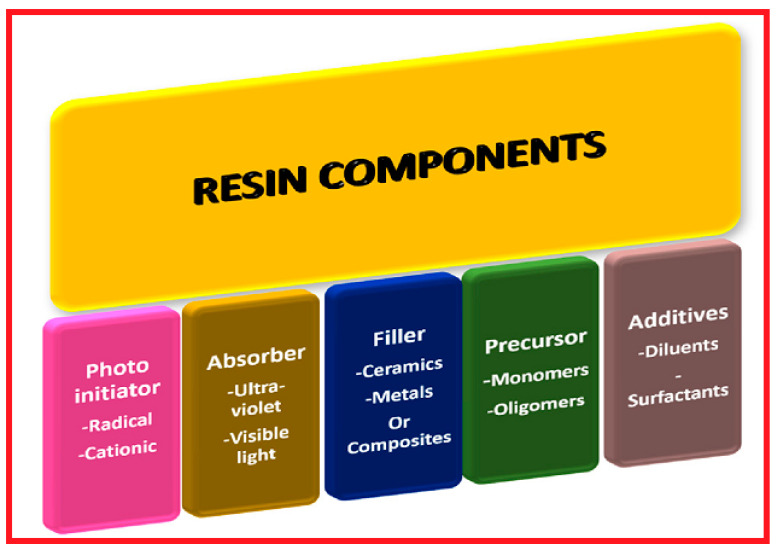
Resin components in stereolithography (SLA) [29].

It is worth mentioning that directed energy deposition (DED), another metal additive manufacturing technology, uses bigger particles and higher layer thicknesses. Han et al. [23] found that the DED of several high-entropy alloys typically has a layer thickness of 100 to 800 µm. SLM, SLS, DMLS, and EBM are all PBF technologies. Heat is utilized to fuse the powdered materials in all of these processes. The distinctions between these methods are in their energy source and powder composition [24]. In contrast to most other additive manufacturing procedures, the powder bed around the created components acts as a support framework, allowing for the manufacture of support-free pieces. Apart from the cost benefits, the elimination of support structures enables more geometric flexibility of design and quicker component manufacturing [25]. SLS is primarily employed in the fabrication of polymers and ceramics, while SLM, DMLS, and EBM are employed exclusively in the fabrication of metals and alloys [26,27]. Stereolithography (SLA) is a procedure that involves selective curing of a photoreactive resin while a platform moves the component after each new layer is formed [21]. In an SLA process, a UV laser is directed in an intended path into the resin reservoir, where the photocurable resin polymerizes, forming a two-dimensional (2D) patterned layer. After curing each layer, the platform is lowered, exposing another uncured resin layer ready for patterning [14,28]. In Figure 5, the resin components of SLA are depicted [29]. SLA printing process is used to fabricate structures such as channels with dimensions less than 100 µm, valves and pumps, and multiplexers for mixing [29].

The process is utilized to fabricate polymer, ceramic, and polymer-ceramic composites with high resolution and accuracy. Nonetheless, limited photoresist materials, residual toxic moieties, and the need for post-processing remain to be obstacles in the biomedical use of SLA scaffolds. In contrast to SLS, SLM employs a high-energy density laser to fully melt the material, hence increasing the component’s mechanical strength and surface quality [30,31]. Powders employed need a homogenous distribution of spherical particles of uniform size. The smaller the size, the more accurate and precise the resolution. When EBM is utilized, a high-vacuum electron-beam gun scans and melts only conductive metal powder (even those with high melting points) materials to produce a part. Scaffolds manufactured with EBM exhibit a significant degree of surface roughness and poor precision [21,31].

**Figure 6:**
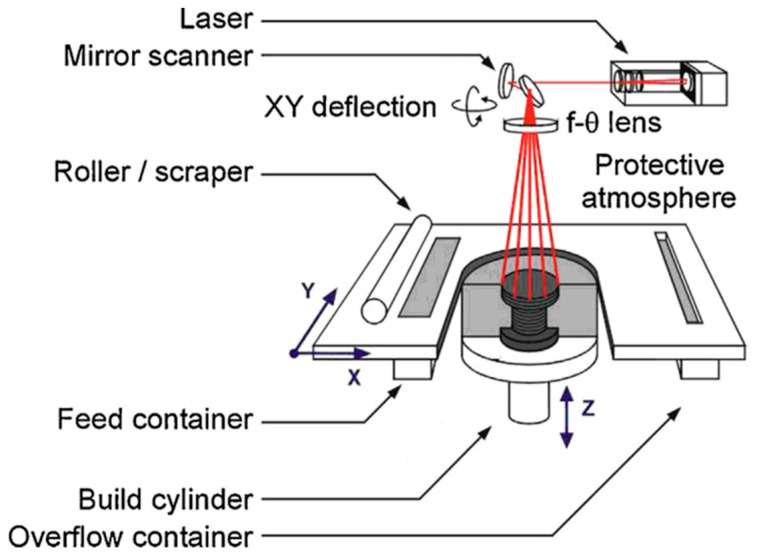
Illustration of SLS process (reprinted with permission) [61].

Selective laser sintering (SLS) is a method for 3D printing that is based on using powders as printing material. In the SLS process, the laser sinters the powder in the powder bed layer-by-layer selectively to generate a 3D structure. Wax, ceramics, metals, and polymers are major SLS topics [31,32,33,34,35,36,37,38,39,40,41,42]. Nylon, i.e., polyamide (PA) [2,34,35,36,37,40,43,44,45,46,47,48,49], and semi-crystalline thermoplastics (polyethylene (PE) [2,50,51,52], polyether ether ketone (PEEK) [2,53,54], and polycaprolactone (PCL) [2,55,56,57,58,59,60]), are among the major polymers used as material in SLS as schematically is shown in Figure 6 [61]. PEEK is an organic thermoplastic polymer that is colorless and belongs to the poly aryl ether ketone (PAEK) family utilized in engineering. The dialkylation of bisphenolate salts results in step-growth polymerization of PEEK polymers. PEEK is a semicrystalline thermoplastic material with good mechanical properties and good chemical resistance and can withstand elevated temperatures. The crystallinity and the mechanical properties of PEEK can be influenced by the manufacturing conditions used to shape it. The tensile strength of PEEK is in the range of 90–100 MPa, and its modulus of elasticity is about 3.6 GPa. The melting point of PEEK is 343 °C, while its glass transition temperature is about 143 °C. Some grades of PEEK can withstand temperatures as high as 250 °C. Between ambient and solidus temperatures, the thermal conductivity of PEEK increases almost linearly. It is used to produce pumps, bearings, piston parts, compressor plate valves, high-performance liquid chromatography (HPLC) columns, and electrical cable insulation, among other things, because of its hardness. PEEK is one of the limited and desired types of plastics that can endure and withstand ultra-high vacuum, making it ideal for many industries like aerospace, shuttles, automotive, electronics, and chemicals. Additionally, PEEK is a novel and innovative biomaterial that is applied in medical implants, like partial replacement skull in neurosurgical applications and spinal fusion devices and reinforcing rods. Moreover, PEEK, also named and known as polyketone, is a thermoplastic with exceptional mechanical properties. In this regard, $T_{s}$ is very close to crystallization curling occurs due to premature crystallization, and the fabricated component is distorted after being released from the powder bed. The premature crystallization can be avoided if the process temperature is a bit higher, but in this case, the temperature is too close to melting, resulting in a lack of precision in the fabricated part. The particles of powder that are close to the laser trace can be stuck on the lateral growth melted surfaces and prevent having desired precision of the manufactured part. Another extreme example of premature crystallization for an unsuitable SLS powder is when crystallization happens too quickly, which in this case, various laser traces are also isolated. Some other essential factors need to be considered for the efficient application of polymer powders in the SLS process, such as melt viscosity, surface tension, and optical properties, apart from the appropriate thermal transitions (T_m_ and T_c_) [38].

PCL is a widely used biopolymer that has great biocompatibility and biodegradability [62]. PCL degrades slowly in human body fluid due to the hydrolysis of ester bonds, producing harmless substances such as carbon dioxide and water [63]. Additionally, due to its superior processing capability, it has been frequently employed as a feedstock material for extrusion-based AM. Another class of synthetic biopolymers based on polyesters include polyglycolic acid (PGA), PLA, and their copolymer poly (lactic-co-glycolic acid) (PLGA), which have attracted considerable interest for biomedical applications. PLA is made from various renewable resources, including corn starch, tapioca roots, and sugarcane [64]. PLA presented slow degradation rate, which resulted in a long in vivo life time that might degrade within 3–5 years. The degradation rate is dependent on the hydrolysis of backbone ester groups and is related to the crystallinity of the PLA, the amount of Mw and its distribution, the morphology of the polymer, the rate of water diffusion into the polymer, and the stereoisomeric content [65]. Kanczler et al. [66] presented the process of porous PLA scaffold fabrication using surface selective laser sintering (SSLS). PLGA, a copolymer of PLA and PGA, is a material that is often used in AM techniques. Lee et al. [67] described a method for producing porous PLGA scaffolds using an indirect 3D printing procedure [68]. Because of the biocompatibility of polydimethylsiloxane (PDMS) polymer, it has been extensively employed in biomedical applications as sensors, medical equipment, and tissue implants. Lee et al. [69] fabricated PDMS surface patterns using fused filament fabrication in additive manufacturing. The findings of this research suggest the use of additive manufacturing to rapidly fabricate scalable structures with anisotropic material characteristics for various applications [69].

The higher strength-to-weight ratio of composite materials over unreinforced polymers or metals is well known and is currently widely used in industry, where the fibers, due to their narrow cross-section, have fewer defects than bulk materials and demonstrate greater strength throughout their lengths [70]. Manufacturing composite material components using additive manufacturing rather than well-established traditional processes such as compression molding provides several benefits, including the ability to create complicated geometric components with thin extended areas and quicker processing [13,71]. The use of composite powders in powder-based AM processes has shown a number of benefits. First, reinforcements may aid manufactured components in achieving increased mechanical properties, fire resistance, thermal conductivity, electrical conductivity, biocompatibility, and piezoelectric characteristics, among other characteristics. Second, the incorporation of nanoparticles such as silica and carbon nanotubes (CNTs) enhances the flowability and light absorption capacity of polymeric powders, facilitating the deposition and fusing processes [72]. Tekinalp et al. [73] found that when the amount of short carbon fiber in the FDM-printed beads rose, the porosity inside the beads increased while the voids between the beads decreased. Matsuzaki et al. [74] investigated composite fiber materials, i.e., fibers infused into PLA for 3D printing, with the goal of improving the mechanical characteristics of the AM components [75]. The influence of modifying the mesostructure of AM-processed CNT-reinforced poly(lactic acid) (PLA) nanocomposites on their mechanical and thermal properties has been investigated [76]. The results showed that the conditions of thermal processing used during AM process had a major effect on the nanocomposites’ crystallization behavior. Therefore, the thermal conditions of AM process should be considered to estimate the mechanical characteristics of the fabricated part. The thermo-mechanical analysis (TMA) above the $T_{g}$ of AM nanocomposites was used for indexing the residual thermal stresses. The bigger dimensional change above $T_{g}$ has been regarded as a measure of the residual stress build-up during AM processes. The structure–property relationship in the AM nanocomposites was analyzed by altering the infill percentage, layer thickness, and infill pattern [76]. Despite having a lower infill percentage in a specified unit of area, the fracture in the specimens with the honeycomb pattern of infill displayed specifications comparable to 0/90, such as similar strands’ in-plain failure at the same cross-section. The partial alignment of extruder strands with the loading direction led to a major increase in stiffness and a slight improvement in tensile strength compared to the specimens with the −45/+45 criss-cross infill pattern. The lower improvement in tensile strength in comparison to stiffness may be because of the higher likelihood of formation of structural faults due to the large intra and trans-raster bonding regions, resulting in an earlier fracture. Nevertheless, in terms of achieving better tensile properties, the infill pattern of honeycomb could be an alternate to the 0/90 pattern [76].

It is worth noting that, in recent years, AM of polymeric composites (and other types of materials) in tissue engineering has become increasingly popular in scientific societies, especially in the medical field [60,77,78,79,80,81,82,83]. It should be mentioned that the significant parameter of “CLTE” (coefficient of linear thermal expansion) is in the following form [80],

(1) $$\mathrm{CLTE}=\frac{∆L}{∆T\times L_{0}}$$

In which, $L_{0}$ represents the primary length of the sample, and ∆L and ∆T are respectively the length and temperature changes. Also, the important relationship between the surface free energy and contact angle has been introduced and presented as the following,

(2) $$\gamma_{SS}=\gamma_{SL}+\gamma_{LL}\times \cos\theta$$

In which, γ represents the surface tension, with the subscripts SS, SL, and LL referring to the solid-solid, solid-liquid, and liquid-liquid interactions, respectively, and θ is the contact angle [80].

Due to the existing 3D porous structures for cell ingrowth and matter transport, facilitating new tissue formation and biodegradation, scaffold-based techniques show significant potential for tissue engineering and regenerative medicine [84,85,86]. As a result, more attention has been paid to designing and developing innovative 3D porous scaffolds for tissue and organ regeneration. 3D printing technology has made significant advances in tissue engineering over the last several decades, enabling the fabrication of patient-specific scaffolds/constructs with specified features [84,85]. The area of tissue engineering and regenerative medicine has been revolutionized by 3D printing technology, which provides unparalleled control, flexibility, speed, and accuracy compared to traditional production processes. A critical but constraining component of 3D printing design and application is selecting appropriate materials for use as biomaterial inks [85,86,87,88,89]. In research [80], the inclusion of various forms of biodegradable and biocompatible iron-based metallic reinforcements (stainless steel 316L and monolithic iron) in PLA-based 3D printed scaffolds was studied through analysis of their properties. Stainless steel 316L and iron powders were used as fillers for manufacturing of 3D printed biodegradable PLA, PLA/316L, and PLA/Iron scaffolds for bone tissue engineering applications employing the fused filament fabrication (FFF) process [80].

**Figure 7:**
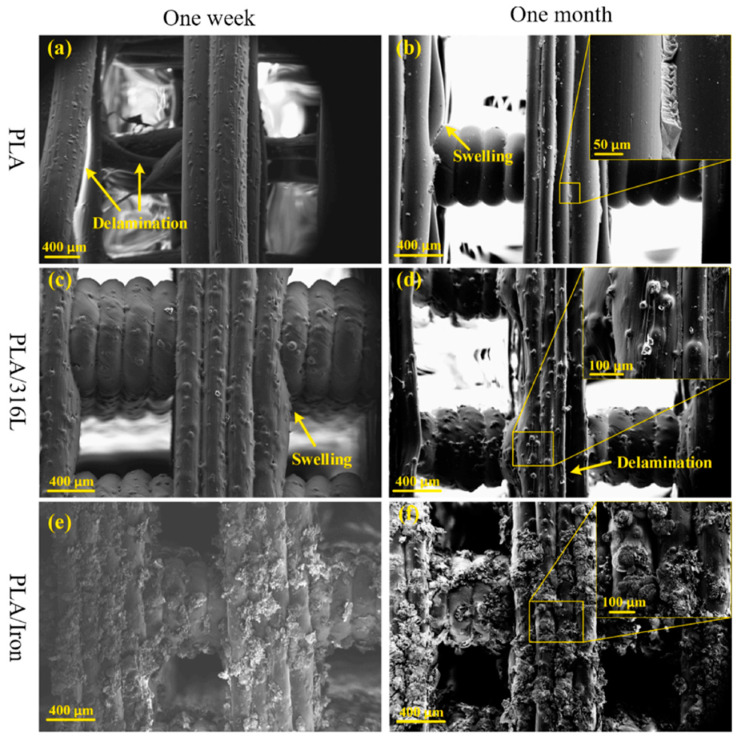
Surface morphology of PLA and PLA-based composite scaffolds after one week and one-month immersion in PBS solution [80].

Figure 7a–f show the surface morphology of monolithic PLA and PLA-based composite specimens after one-week and one-month immersion in PBS solution [80]. It is worth noting that after one week of immersion for the monolithic PLA scaffold specimen, significant delamination between layers was observed. This issue could be because of the hydrolysis mechanism, which weakens the node’s connection. The delamination did not happen in all PLA specimens. However, it suggested that the 3D printed PLA scaffold samples could be failed at the layer’s intersections during the early stages of operation; the swelling in the PLA/316L scaffold specimens after one week of immersion, as well as in the monolithic PLA specimens after one month of immersion, can be seen in Figure 7b,c, respectively. This could be because of PBS’s penetration through voids and other defects, lowering the strut’s modulus and strength. As can be observed in Figure 7e, the corrosion products have covered the surface in the PLA/Iron scaffold specimen. As shown in Figure 7b,d, the surface of PLA and PLA/316L scaffold samples remained undamaged even after one month of immersion, showing their higher resistance to degradation than the PLA/Iron specimens [80]. In the magnified images in Figure 7b,d, a different corrosion product could be seen between layers, which could be the PLA’s hydrolysis product. Peeling off this corrosion product on the PLA/316L scaffold samples can cause delamination between layers, as shown in Figure 7d. However, since no explicit swelling or delamination was found in Figure 7f, it may be concluded that the corrosion agent in the PLA/Iron scaffolds protects the propagation of defects. Furthermore, the scaffold’s voids may be filled by the broad surface area of iron oxide because of the iron’s high Pilling–Bedworth ratio [80].

Wiria et al. [90] presented the use of the SLS method to fabricate a PCL/hydroxyapatite (HA) composite scaffold for tissue engineering applications. In tissue engineering, the combination of these two materials is extremely promising. To achieve superior mechanical characteristics, the laser power, scan speed, and HA loading were adjusted. With 10 wt% of HA and 90 wt% of PCL, a yield stress of 11.54 ± 0.80 MPa and a Young’s modulus of 102.06 ± 11.26 MPa were attained at 2% strain offset. Korpela et al. [91] presented the FDM fabrication of porous PCL/bioglass composite scaffolds. To prepare FDM filaments, 10 wt% bioglass was added to PCL. The incorporation of bioglass particles into porous PCL matrix scaffolds was effective. When bioglass was added, the compressive modulus increased from 104 MPa to 147 MPa [64].

## 2. Some Important AM Flowcharts and Algorithms

**Figure 8:**
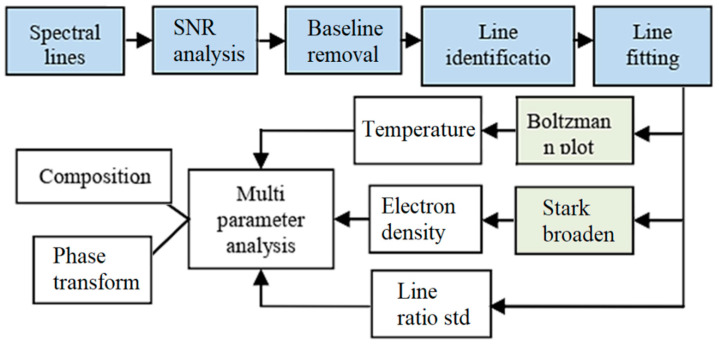
Pre-processing and multi-parameter analysis for monitoring of the composition and phase transformation [92].

**Figure 9:**
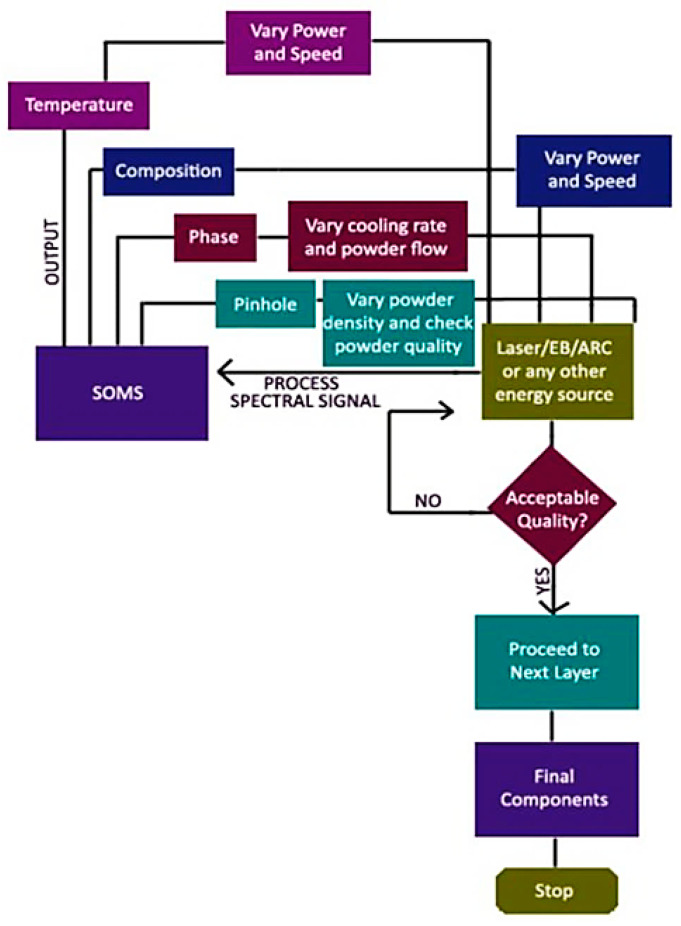
Flowchart for SOMS-based closed-loop control for AM [92].

Some interesting implemented AM flowcharts and algorithms are presented in this section [92,93,94,95,96]. In research [92], the design methodology for the smart metallic additive manufacturing system (s-AMS) was studied. A feasible alternative is in-situ optical diagnostics that can be combined with process management. The two main types of AM used to fabricate components are powder beds such as laser sintering (LS) and pneumatically delivered powder like direct metal deposition (DMD). DMD allows one to deposit different materials at various precisions with a specified height directly based on CAD data, while the feedback loop can control the thermal cycle. Newly developed optical sensors use optical spectra to control the quality and geometry of the part through imaging, and the cooling rate through temperature monitoring, microstructure, and composition. Finally, the mentioned sensors can certify the object that is constructed. Recently researchers have developed a tool for predicting solid-state phase transitions, paving the way for the possibilities for using new process materials and manufacturing routes. This method is highly adaptable, and it is essentially a technology that allows for the creation of a wide range of designs. Seating and manufacturing are also feasible. A signal-to-noise ratio study, baseline reduction, line detection, line de-convolution, and fitting are all part of the plasma spectral line’s pre-processing. In both the visible and UV regions, high-resolution optical emission spectroscopy is used to quantify plasma parameters like spectral line intensity, line ratio, plasma temperature, and electron density, as shown in Figure 8 [92]. The output signal from the smart optical monitoring system (SOMS) has been integrated and used for AM process control to prevent defects and manufacture the components with high quality via the AM [92]. The technique is schematically explained in Figure 9 [92].

**Figure 10:**
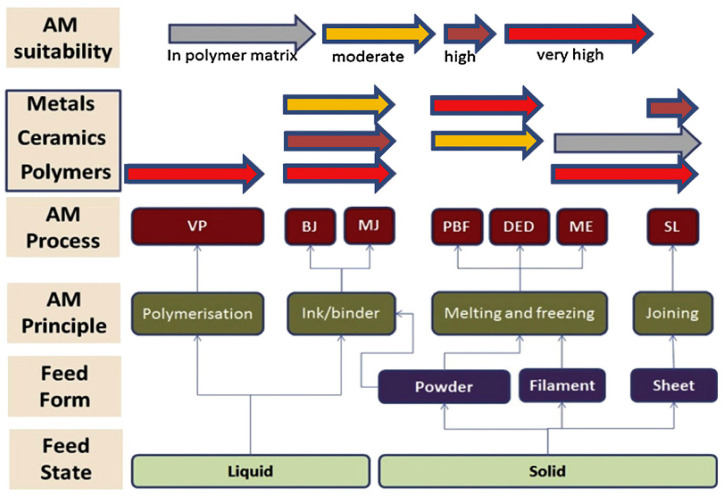
A schematic diagram of the relative suitability of additive manufacturing (AM) of three major types of materials (polymers, ceramics, and metals) in various feed forms and states using ASTM processes: Binder jetting (BJ); directed energy deposition (DED); material extrusion (ME); (4) material jetting (MJ); powder bed fusion (PBF); sheet lamination (SL); and vat photopolymerization (VP) [97].

**Figure 11:**
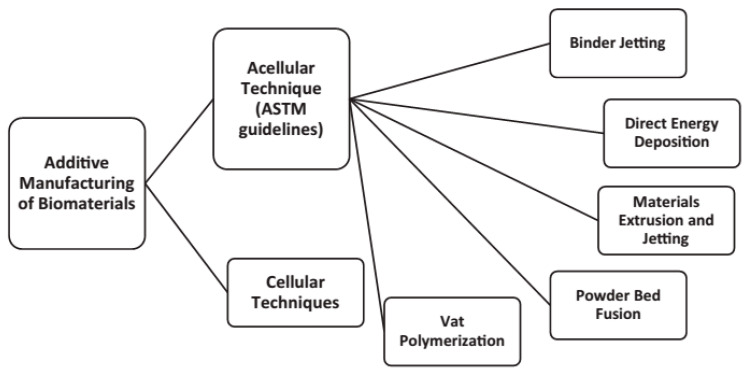
Acellular techniques for the additive manufacturing (AM) of biomaterials [64].

The review paper [93] aimed to keep the research community up to date on the latest technical developments in 3D printed unmanned aerial vehicles (UAV) materials and structures. It addresses the rise of 3D printing for UAVs and shows how it differs from traditional manufacturing routes. In this research, the future of materials and structure for UAVs is explored in detail. In particular, AM technologies’ benefits and potential for improving UAV aerodynamics and structural efficiencies have been highlighted in the study. Issues, obstacles, and potential directions for the understanding of fully printed lightweight UAVs with desired features have been highlighted [93]. Machines and certain AM processes are associated with certain forms, types, and states of materials [94,95,96,97]. The versatile design of AM techniques allows for fast manufacturing without requiring significant changes to the manufacturing setup. Furthermore, the AM techniques are more cost-effective than the traditional manufacturing routes in low-volume production [93]. More significantly, while considering polymer composites, it is determined that only ceramics are acceptable for use in a polymer matrix. Additionally, they were discovered to be compatible with printing procedures such as sheet lamination (SL) and material extrusion (ME), as shown in Figure 10 [97]. In this respect, there are two major groups of AM approaches for biomaterials, including acellular and cellular. The acellular category refers to printing materials that do not include any living cells. Cellular printing entails the printing of living cells in com-bination with other materials. The American Society for Testing and Materials (ASTM) has established a categorization system for the various AM processes [1]. In this regard, Figure 11 illustrates various AM routes for biomaterials schematically [64].

In research [94], the surface flashover characteristics of a non-uniform conductivity insulator fabricated by 3D printing were evaluated. The non-uniform insulator’s conductivity distribution was optimized using a hybrid approach of radial basis function (RBF) and genetic algorithm (GA) neural network models to attain better E-field distribution. The engineered non-uniform insulator was then manufactured using a multi-material FDM 3D printer, while the electrical and thermal properties of the printing materials were investigated. Lastly, under DC voltage, the surface flashover voltages of uniform and conductivity non-uniform insulators were compared in vacuum and sulfur hexafluoride (SF_6_). After that, the mechanism for improved flashover characteristics was addressed [94]. The FFF, like most layer-wise AM processes, suffers from low-performance rates and scalability issues. The concurrent FFF process overcomes these disadvantages by dividing each layer’s processing among multiple extruders working in tandem. The research [95] presented a general toolpath allocation and scheduling methodology to attain this aim. Because of the simplicity with which the composition and microstructure of FFF may be controlled, it was claimed that FFF offers the most potential for producing components for target sectors such as aerospace, biomedical, and automotive. Recent advancements in FFF technology have resulted in the addition of new materials to the existing material library, including metals, high-temperature polymers such as PEEK, and ceramics such as boron nitride. The FFF methodology enables the customization of a range of desired qualities via the use of hybrid materials or alternating concentrations of various components to create functionally graded materials (FGM) [98].

**Figure 12:**
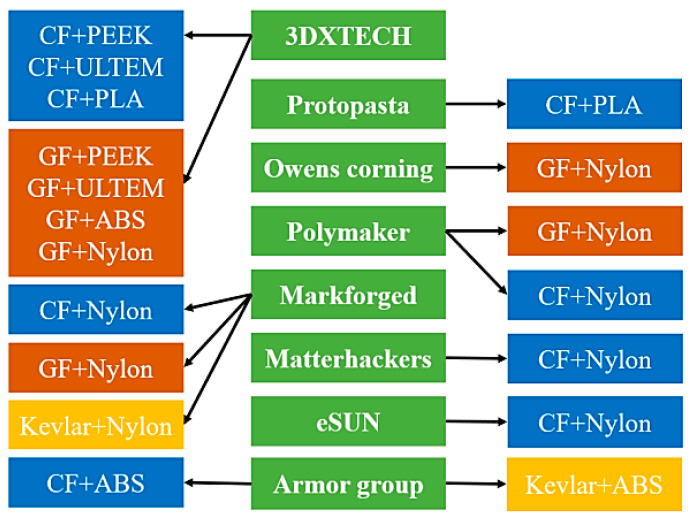
Manufacturers of FDM filament and printer (middle column) and their associated fiber-reinforced composite products. Key fibers reinforcements include carbon fiber (CF), glass fiber (GF) and Kevlar (KF), while popular matrixes are nylon, polylactic acid (PLA), PEEK, and acrylonitrile butadiene styrene (ABS) [96].

**Figure 13:**
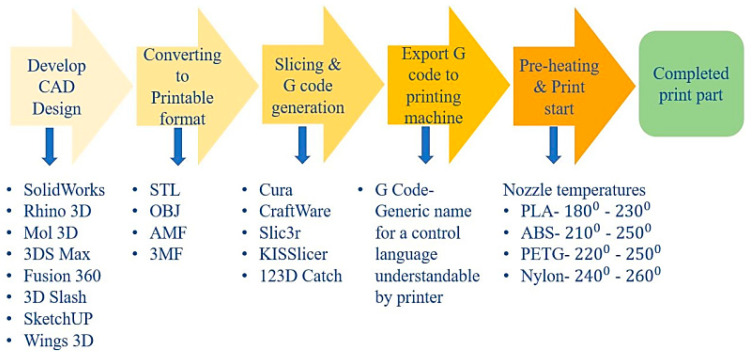
Simplified flowchart of FDM process flowchart, representing different types of software employed in the process and suggested nozzle temperature for some materials [96].

FDM is one of the most common fiber-reinforced composite (FRC) printing techniques. In this method, due to inherent weaknesses in the printed pieces, their efficiency is limited compared to that of other manufacturing methods. As a result, the drive to improve treatment options to address these disadvantages has intensified in recent years. Other research [96,97,98,99,100,101,102,103,104,105,106,107,108,109,110] looked at the effect of the defects on the mechanical efficiency of FRC and subsequently discussed treatment options for removing or minimizing them to improve the functional properties of the fabricated parts. Since FRCs are made up of a polymeric matrix and a short or continuous fiber reinforcement, the analysis will go through the effects of AM parameters such as infill pattern, layer thickness, raster angle, and fiber orientation on both thermoplastic polymers and FRCs printed using FDM technology. The most common defects on printed pieces, such as void formation, surface roughness, and weak fiber-to-matrix bonding, are investigated. The research provides a comprehensive discussion of the efficacy of chemical, heat, laser, and ultrasound therapies in reducing the mentioned disadvantages. The combinations of matrix and fiber materials produced by different manufacturers of filaments are shown in Figure 12 [96]. The filament manufacturer is shown in the middle column, while the fiber and matrix variations are shown in the other two columns. The process of the FDM method is depicted as a simplified flowchart in Figure 13 [96]. FDM is a sluggish printing method that can only be used for materials that have a low melting point. New deposition processes have been developed to allow for more precise control of filler orientation and anisotropy in printed materials. For example, Raney et al. [111] produced a revolving print head that imparts a helical fiber arrangement with spatial control over the helical angle through rotation rate modulation. By applying an external magnetic field and magnetized platelet fillers, Kokkinis et al. [112] established spatial control over filler orientation in printed composites. Collino et al. [113,114] applied acoustic focusing within a deposition channel to concentrate, align, and arrange a wide range of fillers within printed filaments. Gladman et al. [115] printed actuating devices with finely controlled deformation modes using carefully designed print routes and an anisotropic swelling material (as a consequence of filler alignment) [116].

## 3. Mechanical Properties

Mechanical characteristics variation is a major concern in a variety of engineering applications. To address this from an AM process viewpoint, a novel production process for microsurface patterns utilizing fused filament 3D printing was developed and explored the effect of the anisotropic hyperelasticity model on material characteristics [69]. Combining several functional fillers in a polymer matrix enables multifunctional expression [117]. Fiber-reinforced polymer composite AM has much potential for turning 3D printing into a viable manufacturing method. The capability to build complicated functional components with full control over material properties and 3D printing’s unique characteristics, such as high customization combined with added strength from fiber reinforcement, helped 3D printing of FRCs to gain huge attention from a wide range of industries. Automotive, aerospace, biomedical, and electronics are only a few of the industries that have been drawn to 3D printing of FRCs [2]. The chances of defects forming in AM composites are high, resulting in lower strength. Investigating the defect formation mechanism could thus lower the risk of a defect and, as a result, increase performance properties [12]. It could be concluded that using the FFF process to fabricate PLA-based scaffolds reinforced with iron-based powders enhances mechanical properties and dimensional accuracy. Culbreath et al. [118] evaluated the usage of FFF in the fabrication of medical devices by assessing it according to commodity-based implant grade polymers PCL and poly-L-lactic acid (PLLA), as well as a proprietary polymer called Lactoflex. Their findings revealed that mechanical characteristics may be significantly adjusted by varying the quantity of infill and material composition [118]. In general, fiber reinforcement improves mechanical properties such as tensile strength, hardness, fracture toughness, flexural strength, and Young’s modulus. However, the mechanical properties that may be achieved are highly dependent on the density, size, and form of the fiber reinforcement utilized. For instance, it has been found that the inclusion of microsphere resulted in a decrease in tensile strength. The hollow microsphere’s low density is the primary cause for reduced tensile strength [119]. Lu et al. [120] studied the effect of carbon fiber length variation on fracture toughness and flexural strength. The fracture toughness of 2 mm fibers was found to be greater, but the flexural strength of 1 mm fibers was found to be higher. The inclusion of reinforcements was claimed to improve the print quality [119].

Additionally, the mechanical properties of tissue engineering scaffolds are essential, particularly in load-bearing applications. To enhance the mechanical properties of polymer-based scaffolds for biomedical applications, inorganic, organic, and carbon fillers and fibers have been utilized as reinforcements. Among all AM methods, laser-based SLA and digital light processing (DLP) are the best ideals for scaffold manufacturing because of their high resolution and capability to construct complex structures and simplify modifying the scaffold characteristics through liquid resin formulation adjustments [117]. In this regard, for bone tissue engineering application, the PLA/Iron scaffold had much promise. Moreover, a detailed understanding of the relationship between process, thermal activity, and properties could help develop 3D printed polymer-based composite scaffolds. Continuous monitoring for mechanical integrity failure will also be carried out in a simulated body environment [80]. FDM-fabricated low porosity PLA scaffolds with improved mechanical properties were recently studied for bone tissue engineering [121]. Chacón et al. [122] revealed that PLA parts manufactured using the FDM process display anisotropic mechanical behavior that could be altered by varying the build orientation, layer thickness, and feed rate. Chhaya et al. [123] used FDM to manufacture poly(d,l-lactic acid) (PDLLA) scaffolds with a geometry modelled in silico using data from a patient undergoing breast reconstruction surgery [121]. The research on printing parameters in terms of mechanical properties is developing in order to develop 3D printing models with increased strength. Each layer’s bonding is important for the creation of high-strength polymers using FDM. Variation in the raster angle affects the composite’s mechanical characteristics by altering the load transfer between layers [12,124]. As more mobile trans-raster bonding regions formed, the layer thickness increased, resulting in a remarkable reduction in mechanical properties and more porosity due to shrinkage during heating. Furthermore, increasing the percentage of infill enhanced mechanical properties and reduced dimensional changes when heated. Additionally, the pattern of dimensional changes caused by infill percentage variance was extremely non-linear. The infill pattern aided the load transfer inside the mesostructure. According to Ref. [76], a honeycomb-shaped pattern has better thermal and mechanical properties than a regular 45/+45 criss-cross pattern. The variations in print orientation might result in anisotropic properties in the printed component [125]. Research [126] demonstrated that composites with 0◦ fiber orientation displayed superior mechanical properties to composites with 45◦ and 60◦ fiber orientations. Carbon fiber composites with 0◦ fiber orientation had a maximum tensile strength of 165 MPa. According to Mohamed et al. [127], the process parameters involved in product/prototype development using FDM are responsible for the product’s quality and mechanical performance [12]. Carbon-based materials have been studied for decades and have attracted considerable interest in biological applications due to their superior conductivity, unique structure, and mechanical capabilities [117].

Other carbon-based nanomaterials, such as graphene nanoplatelets and CNTs, have been employed to reinforce PEEK in recent years [128]. The printed composite material demonstrated enhanced mechanical and tribological properties, making it suitable for a variety of applications in the aerospace and automotive industries [128,129]. Based on the reports, the composites had homogeneously distributed and aligned CNTs in the PEI matrix, which enhanced the tensile strength; however, during melt compounding, the length of the CNTs was decreased because of the exposure to severe shear stresses [129,130]. Along with pristine PEEK, Chen [131] led research groups that reported printing-reinforced PEEK composites. Chen and coauthors used wet and dry mixing to incorporate graphene nanocomposites into PEEK powder. They reported that the fabricated parts have improved mechanical properties and electrical conductivity [118,131].

Mechanical characteristics of 3D-printed PLA/CNT nanocomposites were explored in this regard, and it was revealed that increasing layer thickness resulted in a considerable loss in mechanical properties, whilst increasing the infill percentage resulted in an increase in mechanical properties since the infill pattern was critical for load transfer within the mesostructured [76]. Furthermore, carbon nanomaterial reinforcements like CNTs and short carbon fibers can be used to enhance the mechanical properties of polymers. Other studies [132,133] demonstrated that the SLS could be used to process CNTs-coated polyamide-12 (PA12) and polyamide-11 (PA11) powders, resulting in embedded CNTs that toughened and reinforced the polymeric matrices simultaneously. Additionally, Yuan et al. [134] created 3D auxetic metamaterials using CNT/PA12 with significant promise for impact protection and cushioning applications. Also, a unique technique has been presented for fabricating high-performance carbon fiber/PA12/epoxy composites by laser sintering and post-infiltration [72]. Moreover, several articles reported decreased mechanical performance at high fiber loadings due to increased porosity, indicating the critical need to address this defect in order to optimize the benefits of fiber inclusion [135,136]. Other research indicated that the addition of continuous fiber reinforcement into the resin composition improved mechanical properties such as tensile strength [119]. Ning et al. [137] reported that incorporating short carbon fibers into FDM-fabricated ABS components reduces the toughness, yield strength, and ductility of the composites and increases the tensile strength, Young’s modulus, flexural stress, flexural toughness, and flexural modulus. Porosity was a major influence in specimens containing 10 wt% carbon fiber [138]. Matsuzaki et al. [74] manufactured continuous carbon-fiber-reinforced polymers by impregnating fibers with plastic filaments within the heated nozzle. Tensile strength was increased significantly when compared to traditional AM composites [138]. Bayush et al. [139] investigated the fiber length distribution of a natural fiber-reinforced polypropylene and concluded that preserving a crucial fiber length and reducing fiber breaking improves the overall compound’s mechanical and dynamic characteristics. Similarly, Gamon et al. [140] demonstrated that longer fibers increase the extruded composite’s flexural strength. Additionally, Inoue et al. [141] revealed that fiber length directly influences the mechanical properties of the mixed composite, as does the screw design’s influence on fiber breakage and dispersion. Furthermore, Hausnerova et al. [142] presented that the shear viscosity and die swell of filled polymers decrease during high shear rate screw extrusion because of fiber length reduction and matrix degradation of the polymer matrix. As a result, it is critical to investigate the influence of fiber aspect ratio distribution on the prediction of elastic characteristics in composites manufactured using large area additive manufacturing (LAAM) [141,143]. The influence of carbon fiber length and weight ratio has been investigated on the mechanical properties of printed ABS resin components using the FDM method [137,144]. With only 5 and 7.5 wt%, an increase in tensile strength and Young’s modulus was attained. The results indicated that longer carbon fibers offer the greatest strength and stiffness. Tian et al. [145] printed carbon-fiber-reinforced polylactide polymer composite components. The fiber content was adjusted during the component printing process by altering the process parameters. For the fiber concentration of 27 wt%, the modulus reached 30 GPa and the flexural strength reached 335 MPa. Similarly, Li et al. [146] studied the mechanical properties of 3D-printed continuous carbon-fiber-reinforced polylactic acid composites [147]. 

AM of large CF-reinforced polymer parts, like aircraft structures or boat hulls, is feasible when existing research on FFF for fiber-reinforced and thermosetting materials is integrated [95]. A volume fraction of 10% has been shown to be the most successful in many experiments utilizing short fiber-reinforced composites. The composite’s mechanical properties are affected by the form of matrix used [96]. A typical post-processing technique for increasing the mechanical performance of FDM fabricated parts is heat treatment, also known as annealing. The gaps between layers are filled when the part is heated, resulting in a smoother surface. The molecular surface tension is decreased when viscosity reduces at the glass transition temperature, resulting in the flowing of the substance on the surface. The substance reflows inside the layers, covering porous regions, and cracks, and providing a staircase effect for a cleaner surface finish and improved mechanical properties. As a consequence, FDM is one of the most efficient routes to fabricate complicated, light-weight components. The production possibilities would be even more promising when its limitations are resolved [96]. The achievement of necessary mechanical properties may be achieved by selecting the appropriate AM method and binder. The future potential of AM in the manufacturing of composites has been emphasized, which opens up new research horizons [119].

## 4. Conclusions

A brief analysis of AM of polymeric composites has been conducted in this mini-review paper based on recent findings. The relevant findings of this mini analysis study are as follows. Depending on the starting material’s state, 3D printing techniques are categorized in liquid, filament or paste, powder, and solid sheet. Layers are produced using UV light-induced polymerization, ink-jet printing, laser melting, extrusion, and other techniques. Polymers were the first materials to be researched in 3D printing, but 3D printing of metals, ceramics, and composite materials to manufacture functional components has recently gained much attention. Using high-power laser and electron-beam-based AM methods, fabricating fully dense metallic parts with mechanical characteristics close to bulk metals has become available [1]. Unlike conventional manufacturing processes, additive AM is not just a tool for shaping; it can also be used to create parts made of multiple materials [64]. As previously discussed, the materials spectrum for AM is still somewhat limited because of the specific requirements of each AM technique. As a result of this constraint, traditional AM polymers are often limited in their suitability for high-performance applications [68]. A significant factor in the long process is the inherent concern about the reliability and reproducibility of AM components [64]. Thus, it is essential to develop high-performance functional materials in order to increase the capability of AM. These capabilities can be realized by designing composite materials that are compatible with the AM method. A composite material is a substance created by mixing two distinct materials with different characteristics [68]. It has been noticed that components created using polymer composites exhibit increased strength and stiffness, as well as significant weight reduction, which has resulted in a significant amount of research being concentrated on material selection and enhancing the characteristics of printed components [148]. The use of these fibers/fillers enhances the mechanical characteristics of AM composites [149]. Reinforcing polymers with fiber/filler has a synergistic influence on the performance and properties of the polymer [150,151]. Due to the complications associated with the use of long and continuous fibers, short and discontinuous fibers are preferred in AM. Cost-effective fiber-reinforced composites may be manufactured using AM [12]. The majority of extrusion-based methods are applicable to the fabrication of short-fiber reinforced polymeric composites. Continuous fibers, on the other hand, may only be combined with thermoplastic or thermoset precursors prior to mechanical extrusion in FFF. The distribution of short fibers and the arrangement of long fibers are designed by the formulation of materials and process patterning, which have a substantial impact on design techniques for optimizing the structural performance of printed components [152]. By printing composites with optimal fiber lengths, easy processing and good mechanical properties can be achieved, expanding the applicability of FDM. The unidirectionally printed components exhibit a considerable degree of anisotropy. For high modulus fibers, printing the continuous fibers at various angles in each layer is challenging due to the isotropic nature of the printed specimen. Additional research on printing isotropic components is required [153]. Carbon fiber and glass fiber are often used reinforcements in AM applications. Numerous investigations have been conducted in both academic and industrial fields on printable fiber-reinforced composites [68] since fiber distribution, fiber orientation, and fiber length influence the mechanical properties of the resulting polymer-based composites [68]. While reinforcing helps enhance the performance of polymer composites, most printed composites still have limited mechanical strength and are unable to satisfy functional requirements. To improve the performance of printed components, further post-processes, including infiltration or consolidation, have been applied [28]. However, some other studies [154,155,156,157,158,159,160,161,162,163,164,165] evaluated additive manufacturing of polymer matrix composites for drug delivery, wound healing, and tissue engineering.

Concisely, the guide outlines the most important factors and their implications for SLS processing. The importance of combining intrinsic and extrinsic properties of polymers to make a suitable polymeric powder for the SLS process is highlighted. Only a specific combination of listed properties has a chance of succeeding, resulting in the availability of fewer commercial materials to date. In the future, SLS technology will necessitate a significant expansion of the polymer powder portfolio, especially for olefin polymers (PP, PE) [38]. However, because of the complicated consolidation behavior and molecular diffusion process involved in sintering, the materials employed in the SLS process are restricted. PCL and PA are now the most extensively utilized laser sintering materials [28]. Additional advancements for 3D printers include increasing printing resolution without increasing printing time or reducing the complexity of the geometry of objects [28]. Despite several constraints, 3D printing technology is advancing at a rapid pace. Numerous published publications and various printed parts in biomedical, aeronautics, electronics, and automotive industries testify to this advancement. 3D printers are not yet capable of handling the volume needs of the industry. This approach must continue to improve to compete with more established production processes [147]. Cutting-edge advances in four-dimensional (4D) printing, nano/microfabrication, and smart drug delivery are revolutionizing the field of biomedical AM. These, in conjunction with the development of new materials engineering tools, and the rising automation and digital control of design and manufacturing processes, with regard to the manufactured products’ resultant characteristics, are predicted to have a great impact on the biomedical industry soon [121].

## Figures and Tables

**Table 1:** Description of the scientific symbols.

Symbol	Description, (Unit), Address
$L_{0}$	The initial length of the specimen, (m), Equation (1)
ΔL	Change in length, (m), Equation (1)
ΔT	Temperature changes, (K), Equation (1)
γ	Surface tension, (Pa), Equation (2)
θ	Contact angle, (degree), Equation (2)
$T_{s}$	Sintering Temperature
*T_c_*	Crystallization temperature
*T_m_*	Melting temperature

**Table 2:** Abbreviation used in the article.

Abbreviations		Abbreviations	
3D	Three-dimensional	PAEK	Poly aryl ether ketone
ABS	Acrylonitrile butadiene styrene	PBF	Powder bed fusion
AM	Additive manufacturing	PBS	Phosphate-buffered saline
CAD	Computer-aided design	PCL	Polycaprolactone
CF	Carbon fiber	PDMS	Polydimethylsiloxane
CLTE	Coefficient of linear thermal expansion	PE	Polyethylene
CNT	Carbon nanotubes	PEEK	Polyether ether ketone
CTE	Coefficient of thermal expansion	PLA	Polylactic acid
DED	Directed energy deposition	PLLA	Poly-L-lactic acid
DLP	Digital light processing	PLGA	poly(lactic-co-glycolic acid)
DMD	Direct metal deposition	PMC	Polymer matrix composite
DMLS	Direct metal laser sintering	PP	Polypropylene
DRAM	Distributed recycling for additive manufacturing	RBF	Radial basis function
EBM	Electron beam melting	s-AMS	Smart metallic additive manufacturing System
FDM	Fused deposition modeling	SEM	Scanning electron microscope
FFF	Fused filament fabrication	SF_6_	Sulfur hexafluoride
FRC	Fiber-reinforced composites	SL	Solid–liquid (interactions)
GA	Genetic algorithm	SLA	Stereolithography
GF	Glass fiber	SLM	Selective laser melting
HPLC	High-performance liquid chromatography	SLS	Selective laser sintering
HA	Hydroxyapatite	SOMS	Smart optical monitoring system
KF	Kevlar fiber	SS	Solid–solid (interactions)
LL	Liquid–liquid (interactions)	TMA	Thermo-mechanical analysis
LPT	Longest processing time first	UAV	Unmanned aerial vehicle
LS	Laser sintering	UV	Ultraviolet
PA	Polyamide		

## Data Availability

All data provided in the present manuscript are available to whom it may concern.
